# Broad neutralizing activity of a human monoclonal antibody against H7N9 strains from 2013 to 2017

**DOI:** 10.1038/s41426-018-0182-2

**Published:** 2018-11-14

**Authors:** Cong Chen, Zuliang Liu, Liguo Liu, Yan Xiao, Jianmin Wang, Qi Jin

**Affiliations:** 1 MOH Key Laboratory of Systems Biology of Pathogens, Institute of Pathogen Biology, Chinese Academy of Medical Sciences & Peking Union Medical College, Beijing, China; 2 Collaborative Innovation Center for Diagnosis and Treatment of Infectious Diseases, Hangzhou, China

## Abstract

H7N9 influenza virus has been circulating among humans for five epidemic waves since it was first isolated in 2013 in China. The recent increase in H7N9 infections during the fifth outbreak in China has caused concerns of a possible pandemic. In this study, we describe a previously characterized human monoclonal antibody, HNIgGA6, obtained by isolating rearranged heavy-chain and light-chain genes from patients who had recovered from H7N9 infections. HNIgGA6 recognized multiple HAs and neutralized the infectivity of 11 out of the 12 H7N9 strains tested, as well as three emerging HPAI H7N9 isolates. The only resistant strain was A/Shanghai/1/2013 (H7N9-SH1), which carries the avian receptor alleles 186V and 226Q in the sialic acid-binding pocket. The mAb broadly neutralized divergent H7N9 strains from 2013 to 2017 and represents a potential alternative treatment for H7N9 interventions.

## Introduction

The H7N9 influenza virus was first isolated in southeastern China in early 2013^1^. Since then, five epidemic waves have been reported that have caused 1258 human infections, with ~40% resulting in death^2^. H7N9 causes severe respiratory distress syndrome in patients and is frequently associated with secondary bacterial pneumonia caused by multidrug-resistant *Acinetobacter baumannii* and *Klebsiella pneumonia*^3^. Noticeable dysbiosis of the oropharyngeal microbiome in H7N9 patients has also been observed^4^. During the fifth epidemic alone, 688 human infections were confirmed, making it the largest H7N9 epidemic to date. Although no sustained human-to-human transmission of the H7N9 virus was confirmed, limited human-to-human transmission has been observed^2^. Because H7N9 viruses are able to be transmitted by the airborne route between ferrets^5^ and can infect and replicate in the human lower airways^6^, they have the potential for efficient human-to-human transmission and are an increasing pandemic threat. Furthermore, unlike H5N1, a high pathogenicity avian influenza (HPAI) virus that causes severe disease in birds and poultry, most of the currently isolated H7N9 viruses are low pathogenicity avian influenza (LPAI) viruses that typically elicit no observable signs of disease in birds after viral infection. Early warning and disease control for an H7N9 pandemic may be extremely difficult, although HPAI H7N9 viruses that are more pathogenic in birds and mammals have recently emerged^7–9^. H7N9 cases have spread to 22 provinces and municipalities in mainland China^2^. Due to its ability to more readily be transmitted from birds to humans, avian-origin H7N9 has raised concerns regarding its potential for increasing the possibility of a pandemic. Thus, it is prudent to conduct clinical trials to identify an effective treatment against H7N9 influenza.

As a typical member of influenza A viruses, H7N9 is classified into subtypes based on the two major surface glycoproteins, haemagglutinin (HA), and neuraminidase (NA), which are responsible for viral recognition, attachment to the cellular receptor and viral release. HA and NA are ideal targets for antiviral drug design. NA inhibitors, including oseltamivir and zanamivir, are currently the primary therapeutic treatment against H7N9 infection in clinical settings^10,11^. However, because of the emergence of escape mutants that are resistant to oseltamivir or zanamivir or even both^10,11^, alternative treatment options for human H7N9 infection are urgently needed. Vaccination is the most effective intervention against seasonal influenza. It has been demonstrated that the H7N9 vaccine is able to induce the production of both neutralizing and nonneutralizing antibodies in humans^12^, and an inactivated H7N9 vaccine has entered clinical trials^13^. It was very interesting and encouraging to discover that some nonneutralizing antibodies could also protect mice from H7N9 infection through Fc–FcgR interactions^12^. Vaccination with seasonal H3N2 strains was also shown to elicit H7 cross-reactive antibodies, although the level of serum protection in the general population remains to be determined^14^. However, in the event of a pandemic outbreak, massive vaccinations against an emerging virus cannot promptly achieve herd immunity. Limited by antiviral drug resistance, neutralizing therapeutic antibodies are considered to be a potentially effective treatment for influenza infections. Passive immunotherapy using convalescent plasma from patients to treat H5N1 and H1N1 infections has achieved encouraging results and has reduced mortality^15–17^. However, the large-scale production of antiserum is not possible in response to an emergency epidemic. The production of neutralizing monoclonal antibodies (mAbs) would provide a feasible solution to this problem. Recent studies have characterized several neutralizing antibodies from human donors that target different epitopes on viral HA proteins, such as CT149^18^, H7.167^19^, m826^20^, HNIgGD5^21^, and HNIgGA6^22^, all of which represent potential interventions in the event of an H7N9 pandemic.

HNIgGA6 was isolated by our lab by isolating rearranged heavy-chain and light-chain genes from human survivors who had recovered from A/Anhui/1/2013 (H7N9-AH) infections. The antibody exhibited potent neutralizing activity against H7N9 influenza in vitro and in vivo. In this study, we determined the breadth of the effectiveness of HNIgGA6 against divergent H7N9 strains isolated from March 2013 to January 2017, as well as against three HPAI H7N9 variants. A series of representative viral isolates were tested in pseudovirus-based neutralization assays. We report that HNIgGA6 can neutralize the most prevalent H7N9 strains. Other than an early A/Shanghai/1/2013 (H7N9-SH1) isolate, all prevalent H7N9 strains from 2013 to 2017 could be neutralized by this antibody.

## Results

### Polygenetic analyses of H7N9 HA

**Fig. 1:**
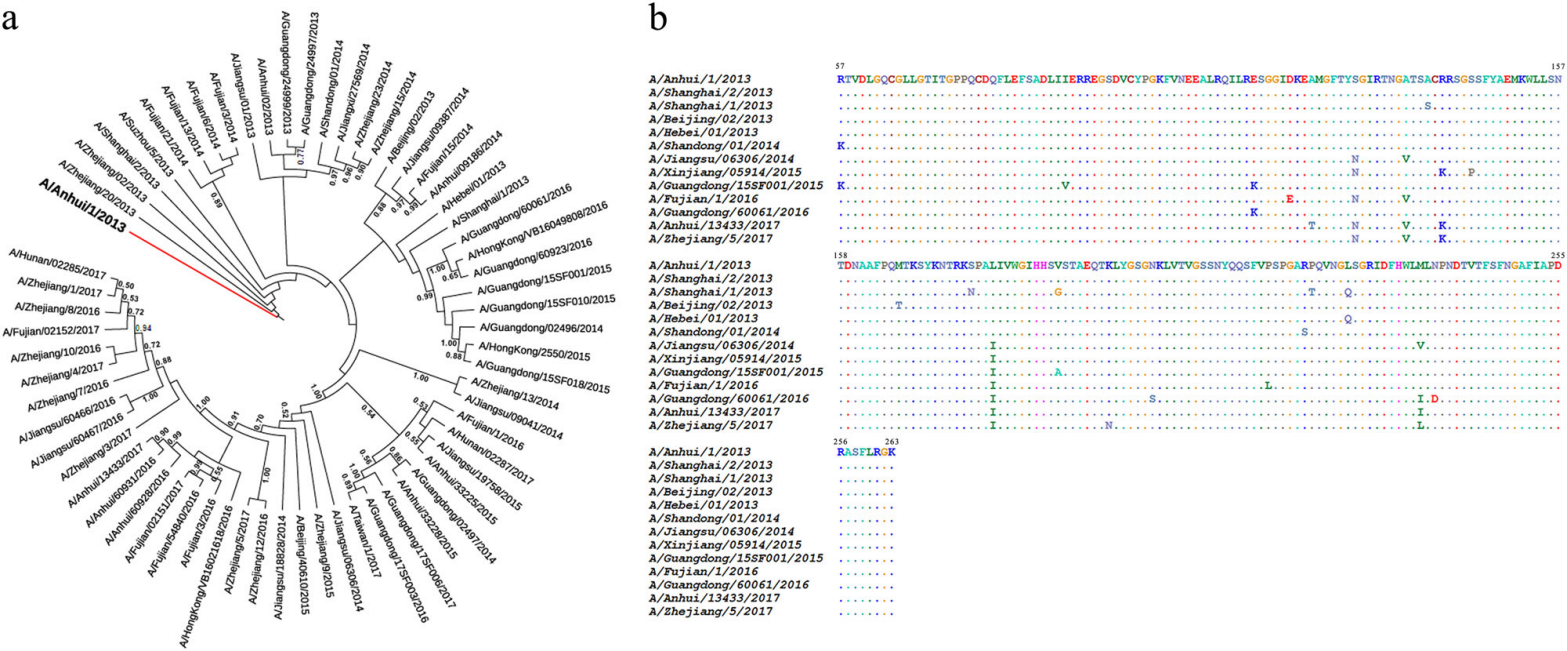
Evolution of H7 HAs. **a** Phylogenetic analysis of the HA genes of representative H7N9 viruses collected since 2013, with an emphasis on the 2013–2017 period. HA sequences were collected from GISAID, the Global Initiative on Sharing All Influenza Data (http://platform.gisaid.org/epi3). The evolutionary tree was inferred using the neighbor-joining method and was generated in MEGA7. The candidate vaccine strain (A/Anhui/1/2013) is shown in the red line, and the viruses used in this study are shown in the blue lines. **b** Sequence alignment of the HA1 domain for the 12 selected H7 HAs. The “.” symbol indicates sequence identity with the H7N9 of A/Anhui/1/2013 strain

To trace the evolution of the H7 HAs in the five epidemic waves, we randomly selected 65 HA genes of human H7N9 viruses from April 2013 to January 2017 and conducted polygenetic analyses. As shown in Fig. 1a, the results confirmed that all the human H7s descended from the same ancestor. Most of the HAs from 2013 to 2014 were very similar and formed a cluster. However, after the initial outbreak, a broad dissemination of the virus was observed that indicated the occurrence of more frequent amino acid changes in HA. Previously, we reported that an H7N9-neutralizing antibody, HNIgGA6, recognized the viral receptor binding site (RBS) on HA1^23^. To evaluate potential antigenic drift, we performed an alignment of 12 randomly selected HA1 sequences and assessed the amino acid substitutions in the viral RBS. As shown in Fig. 1b, many amino acid changes occurred, most of which were in the hypervariable region around the receptor-binding pocket.

### HNIgGA6 binds most of the assayed HAs

**Fig. 2:**
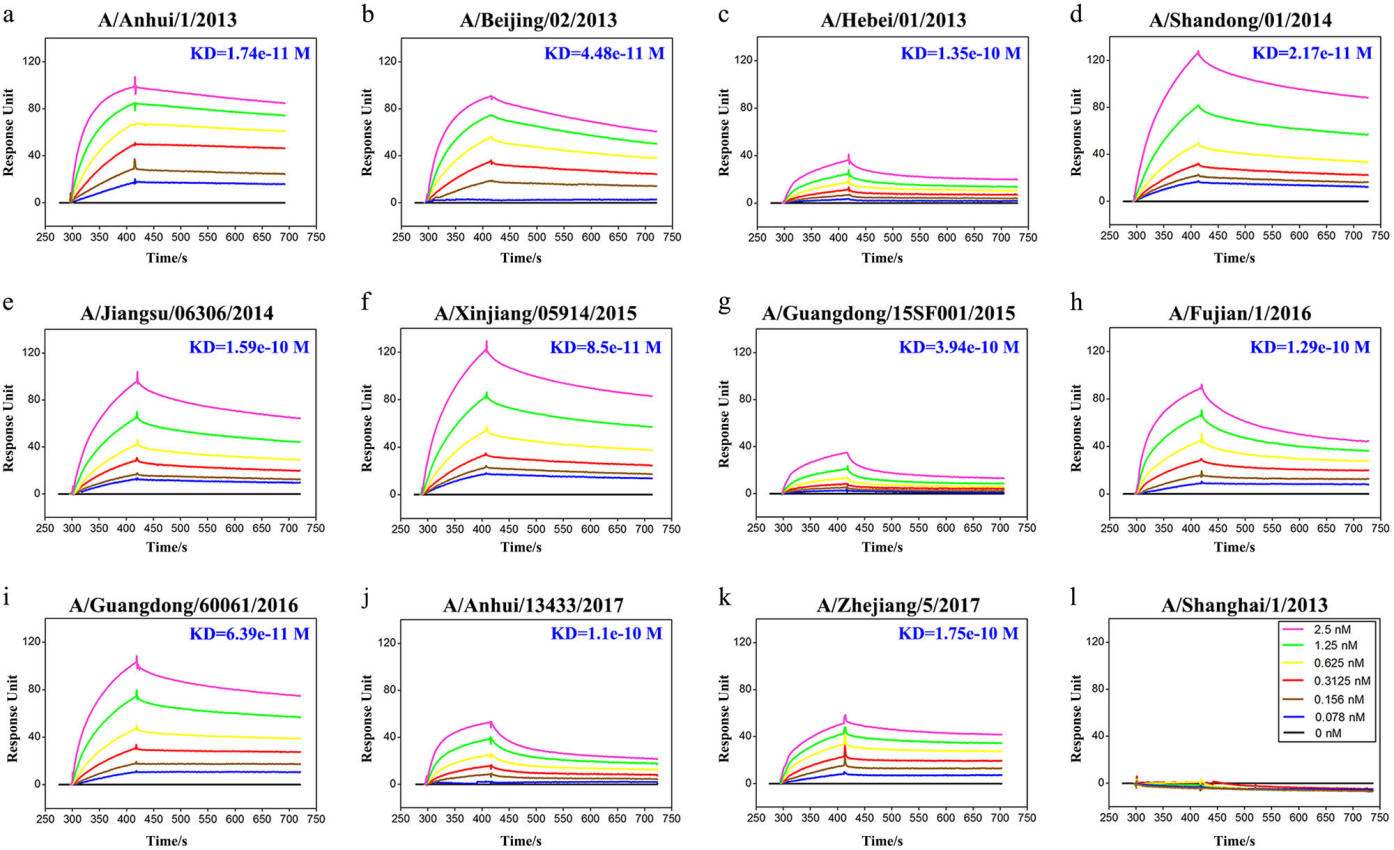
High binding affinity of the human mAb HINgGA6 to H7N9 HA strains. BIAcore plots showing binding of viral HA1 to the HINgGA6 mAb. The mAb was immobilized on a CM5 chip, and a series of concentrations of HA1 were flowed over the immobilized mAb. Binding affinity (*K*_D_) values were calculated using a steady-state affinity model produced with the BIAcore 3000 analysis software

To determine the breadth of activity for HNIgGA6, its binding affinity was tested against a panel of HA1s. Of the 12 strains tested (Fig. 2), HNIgGA6 only failed to bind to the HA1 of H7N9-SH1, whereas it bound the other 11 strains with a high affinity (*K*_D_ values from 1.1e−10 to 6.39e−11 M). Thus, the mAb HNIgGA6 presents strikingly strong, broad binding activity, suggesting it has a broad neutralizing activity against divergent H7N9 strains.

### Mutations at V186G and L226Q result in antigenic drift of H7N9-SH1

**Fig. 3:**
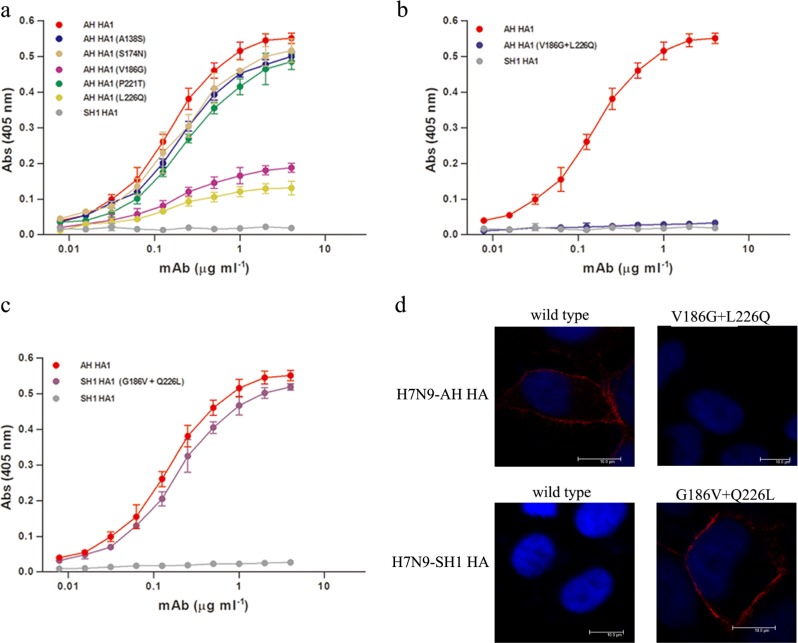
Molecular determination for antigenic drift of H7N9-SH1. **a** Binding of H7N9-AH HA mutants (A138S, S174N, V186G, P221T, and L226Q) to HNIgGA6 was measured by ELISA. **b** Dual mutations (V186G and L226Q) resulted in a complete loss of binding of the antibody to the HA of H7N9-AH. **c** The mutations G186V and Q226L restored the ability of the mAb to bind the HA of H7N9-SH1. **d** Viral HA and the mutated HA proteins were expressed in HeLa cells and detected via IFA

Comparing the HA1 of H7N9-AH with that of H7N9-SH1, five amino acid substitutions occurred, including A138S, S174N, V186G, P221T, and L226Q. To assess their roles in the antigenic drift of the virus, we constructed five mutants of H7N9-AH HA1 such that each carried one of the five corresponding amino acids present in H7N9-SH1. All the mutants were confirmed, and as shown in Fig. 3a, all five mutants retained the ability to bind HNIgGA6. Nevertheless, the V186G or L226Q mutations led to a noticeably reduced affinity. Dual mutations at V186G and L226Q resulted in complete loss of binding to the mAb (Fig. 3b). In contrast, when 186G and 226Q in H7N9-SH1 were reversely mutated to V186 and L226, the mutants were restored in their ability to bind HNIgGA6 (Fig. 3c). These findings were further confirmed with full-length viral HA by immunofluorescence assay (IFA) detection. As expected, the HA of H7N9-AH but not H7N9-SH1 was recognized by HNIgGA6 (Fig. 3d). The V186G and L226Q mutations disrupted binding between the H7N9-AH HA and the mAb, whereas binding was observed when both G186V and Q226L were introduced into the HA of H7N9-SH1.

### Broad neutralizing activity of HNIgGA6

**Fig. 4:**
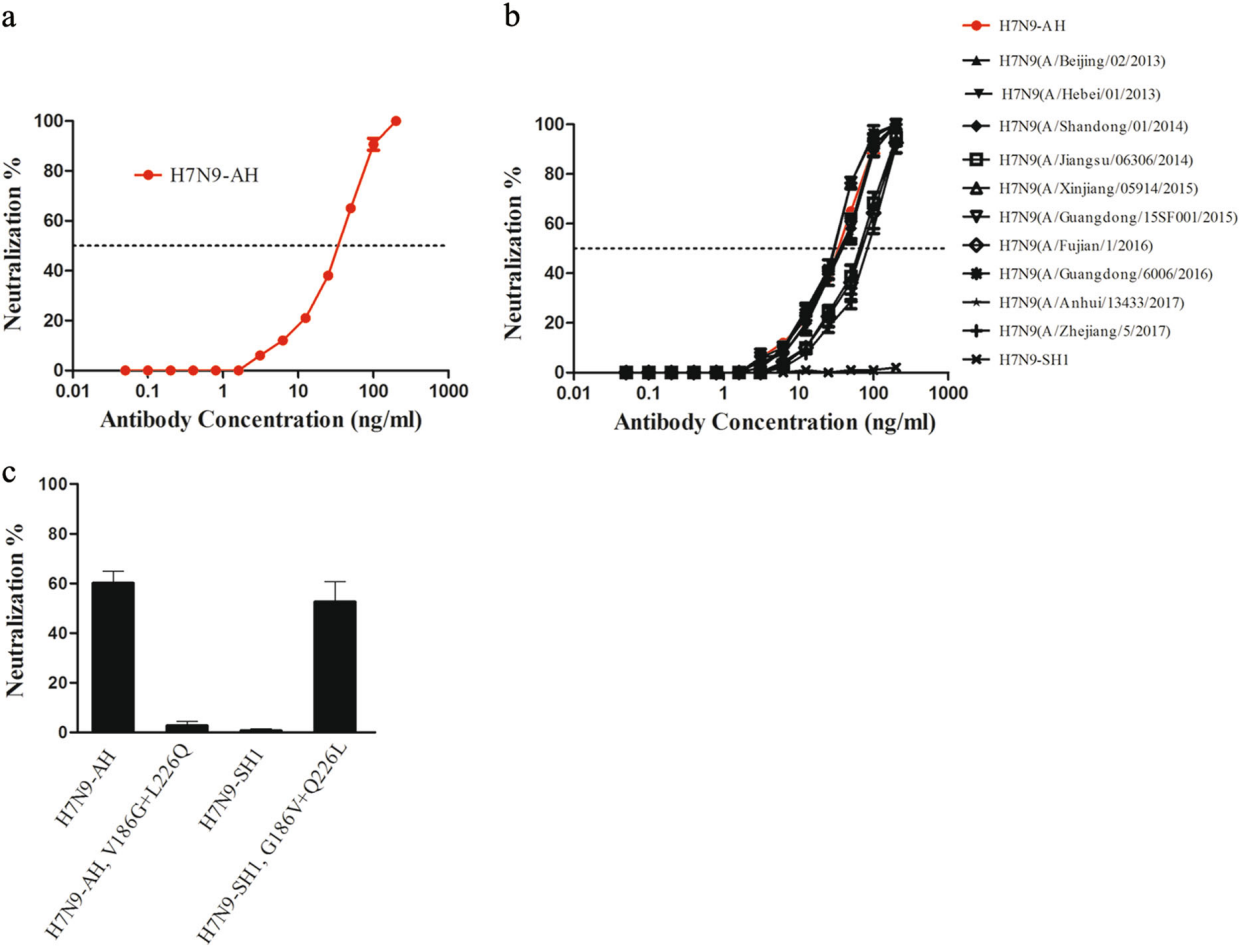
Broad neutralization of the avian influenza A/H7N9 by the human mAb HINgGA6. **a** HINgGA6 neutralized H7N9-AH1 pseudoviruses in a dose-dependent manner. An anti-HIV human IgG was used as a negative control. **b** HINgGA6 neutralized divergent H7N9 pseudoviruses. **c** Pseudoviruses carrying the indicated amino acid mutations were generated. Neutralization assay with 50 ng/ml HINgGA6 was performed using MDCK cells

HNIgGA6 was previously shown to neutralize H7N9-AH and -SH2 influenza viruses in cells and in BALB/c mice^22^. In this study, to avoid the lethal pathogenicity of the live H7N9 virus, we took advantage of a pseudovirus-based neutralization assay to evaluate the neutralizing activity of HNIgGA6. Pseudoviruses expressing the NA of H7N9-AH and divergent HAs were produced in 293T cells, and the neutralizing activity of HNIgGA6 was tested on susceptible MDCK cells. As in the neutralization assay with live H7N9-AH viruses, HNIgGA6 neutralized the H7N9-AH pseudovirus in a dose-dependent manner with an IC_50_ of 41.66 ng/ml (Fig. 4a). The neutralizing activity of HNIgGA6 against divergent H7N9 strains was also tested. As expected, although the H7N9-SH1 strain was resistant to HNIgGA6, the antibody could neutralize the other 11 strains with estimated IC_50_ values from 36.53 to 63.47 ng/ml (Fig. 4b). The V186G and L226Q mutations led to antigenic drift for viral HA1 and the full-length HA protein, and the ability of the mutations to confer resistance against the mAb was also determined using pseudovirus entry assay. As shown in Fig. 4c, when V186G and L226Q were present in H7N9-AH, the pseudovirus successfully escaped from HNIgGA6 pressure. In contrast, when G186 and Q226 were replaced by V186 and L226, the mutated H7N9-SH1 was neutralized the mAb. Taken together, these results confirmed that V186G and L226Q were responsible for antigenic drift in H7N9-SH1.

### HNIgGA6 neutralizes the emerging HPAI H7N9 strains

**Fig. 5:**
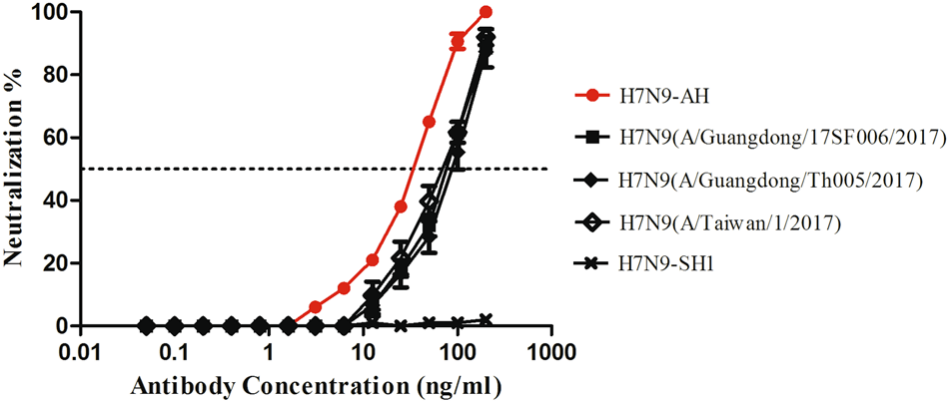
HNIgGA6 neutralizes the emerging HPAI H7N9 strains. Pseudoviruses of three HPAI H7N9 variants (A/Guangdong/17SF006/2017, A/Guangdong/Th005/2017, and A/Taiwan/1/2017) were generated. Neutralization assays were performed using MDCK cells

H7N9 viruses have remained LPAI viruses in poultry since their first appearance in 2013. However, human infections with HPAI H7N9 viruses were reported in February 2017^7,9^. A multibasic cleavage site motif in viral HA was identified that facilitates HPAI H7N9 virus replication in avian species. To assess the neutralizing activity of HNIgGA6 against HPAI H7N9, pseudoviruses expressing the HA of three HPAI H7N9 isolates (A/Guangdong/17SF006/2017, A/Guangdong/Th005/2017, and A/Taiwan/1/2017) and the NA of H7N9-AH were generated. As shown in Fig. 5, HNIgGA6 was capable of neutralizing all three variants with IC_50_ values of ~85 ng/ml.

## Discussion

The avian influenza A H7N9 virus continues to be a serious threat to public health and has raised concerns of a potential pandemic. Thus, it is necessary to develop vaccines and antiviral drugs as well as neutralizing antibodies for the prevention and control of fatal H7N9 infections in humans. In the present study, we characterized a previously isolated human H7N9-neutralizing antibody (HNIgGA6) and determined the breadth of its neutralizing activity.

The significant role of neutralizing antibodies in protecting humans against viral infections has been well demonstrated and is widely accepted. Although palivizumab (anti-respiratory syncytial virus) is currently the only licensed monoclonal antibody, many have been identified and are being explored as therapeutics against a number of viruses, including human immunodeficiency virus-1 (HIV-1)^24,25^, Zika virus^26–28^, Middle East respiratory syndrome coronavirus (MERS-CoV)^29–32^, Ebola virus^33,34^, and influenza virus^20,35,36^. Because it is present on the surface of the influenza virion, the HA glycoprotein is the primary target for developing neutralizing antibodies. However, the high variability of viral HA makes it difficult to develop a highly cross-reactive neutralizing antibody. This is especially true since viral HA is able to continually evolve while maintaining surface sialic acid receptor binding capability, which leads to escape from the antibody response and is known as antigenic drift^37,38^.

Although different HAs display unique structural features, the sialic acid-binding pocket on the HA RBS is highly conserved for the recognition of the common receptor. A few RBS-directed antibodies have successfully achieved modest cross-reactivity^36,39,40^ by interposing a smaller footprint into the receptor binding groove. However, most RBS-directed antibodies have exhibited a limited breadth in their neutralization activity, including 2D1^41^ and 5J8^42^, due to extra contacts with variable residues in the periphery of the conserved pocket. In this study, we tested the binding affinity of HNIgGA6 for 12 different HAs of prevalent H7N9 strains isolated from April 2013 to January 2017. H7N9 HA has been rapidly evolving (Fig. 1a), which is consistent with the previous result that H7 HA is continually mutating at a high rate^43^. Our results showed that HNIgGA6 utilizes avidity to extend its breadth of recognition and to increase its affinity against a large portion of divergent HA strains (11 out of 12), including the A143V/R148K variant, which has become the dominant circulating strain in the fifth wave^43^. HNIgGA6 exhibited a loss of binding to the HA variant on the H7N9-SH1 strain, an earlier isolate from the 2013 Shanghai epidemic carrying an avian H7 signature^1^. The preference for avian α−2,3-linked sialic acid cellular receptors rather than human α−2,6-linked sialic acids receptors limited its transmission to humans, and the SH1 strain was rarely observed in latter outbreaks. Two amino acids substitutions (V186G and L226Q) were responsible for the preference of different cellular receptors, as well as the antigenic drift of H7N9-SH1 (Fig. 3). Although HNIgGA6 recognized 11 other HAs, the observed binding affinities were not completely consistent. An approximately tenfold decrease in binding affinity was observed for A/Hebei/01/2013 and A/Guangdong/15SF001/2015 carrying the L226Q and V186A mutations, respectively, indicating important roles of V186 and L226 in binding HNIgGA6. The reason for the attenuated binding affinity to A/Jiangsu/06306/2014, A/Fujian/1/2016, A/Anhui/13433/2017, and A/Zhejiang/5/2017 remains to be determined. However, the A135V substitution in the RBS that the four strains share in common is surely worth studying further.

All H7N9 pseudoviruses, including three emerging HPAI H7N9 isolates, were neutralized by HNIgGA6 with the exception of H7N9-SH1 (Figs. 3b and 5). However, because the pseudotyped virus particle entry assays may be overly sensitive, an accurate assessment of the in vitro and in vivo neutralizing activity of HNIgGA6 using live H7N9 viruses is still needed. This is especially necessary since mutations on other genes besides HA are also associated with viral replication capacity, pathogenicity, and transmissibility. The E627K mutation in the polymerase basic 2 (PB2) gene enhances H7N9 replication in mammals^44^. The K526R mutation in PB2, which was identified in a HPAI H7N9 (A/Taiwan/1/2017) strain, also contributes to improve replication efficiency together with 627K^45^. The NA R292K mutation confers resistance to both oseltamivir and peramivir^46^. Nevertheless, based on genetic and phylogenetic analyses, the WHO has recommended that H7N9-AH may serve as a viable vaccine candidate for broad protection against H7^47,48^. A comprehensive survey of common resistance mutations among the many strains tested here suggests that mAb HNIgGA6 may be a useful template for a therapeutic antibody.

Genetically and antigenically novel H7N9 influenza viral strains will continue to emerge due to the genetic nature and broad host range of this virus. In the absence of an effective vaccine, direct administration of H7N9-neutralizing antibodies could be used as an intervention to prevent H7N9 infections in humans. This approach could be especially helpful for those at high risk for contracting H7N9. When administered prophylactically, HNIgGA6 conferred 100% protection in virus-infected mice^22^, supporting this rationale. Alternatively, the RBS-directed neutralizing antibody HNIgGA6 can be used together with other antiviral drugs (e.g., oseltamivir and zanamivir) to control the production of the escape mutants that limit the effectiveness of these NA inhibitors^10,11^.

## Materials and methods

### Phylogenetic tree construction

A total of 65 influenza A H7N9 strains isolated during the 2013–2017 seasons throughout China were selected and genetically characterized. The retrieved full-length HA amino acid sequences of influenza A H7N9 viruses were aligned with the reference sequences available in the GISAID database (http://platform.gisaid.org/) by “ClustalW” using Molecular Evolutionary Genetic Analysis (MEGA) version 7. Multiple alignment sites with gaps in any of the sequences were excluded. Phylogenetic trees were generated by the maximum-likelihood method in MEGA7 with 1000 bootstrap replicates. The constructed phylogenetic trees were analyzed for possible geographical linkages.

### Protein expression and purification

The globular head HA1 protein (residues 47 to 322, based on H3 numbering) was expressed using the Bac-to-Bac baculovirus expression system (Invitrogen). Briefly, the HA1 coding sequence was amplified by PCR and inserted into the pFastBacI vector (Invitrogen). To facilitate protein secretion and purification, an N-terminal gp67 signal peptide and a C-terminal 6 × His-tag were fused to the HA1 gene. The recombinant bacmid was transfected into Sf21 insect cells, and the protein was purified by nickel-nitrilotriacetic acid (Ni-NTA) affinity chromatography followed by size exclusion chromatography on a Superdex 200 column (GE Health Care). The HA and NA genes of H7N9-AH are stocked by our lab. The remaining 11 viral HA1 genes were obtained by site-directed mutagenesis in the RBS with the H7N9-AH strain according to the gene alignment results in Fig. 1b.

### Production of human IgG1 protein

Human IgG1 protein for the mAb HNIgGA6 was purified as described previously. The heavy-chain and light-chain genes were cloned into the antibody expression vector and expressed by transient transfection in HEK293F cells using Lipofectamine 2000 (Invitrogen). The cell culture was collected 72 h later and was centrifuged to remove cell debris. Human IgG1 protein was then purified by affinity chromatography using Protein A agarose (TransGen Biotech) and was further purified by size exclusion chromatography. The protein concentration was measured spectrophotometrically (GE Healthcare).

### Enzyme linked immunosorbent assay

The binding of HNIgGA6 to viral HA1 proteins was measured by enzyme linked immunosorbent assay (ELISA). The 96-well plates were coated overnight at 4 °C with viral HA1 protein (50 ng per well). Serial four-fold dilutions of antibodies (beginning with 100 ng) in PBS were made and assayed for binding to recombinant HA1 proteins. After washing, bound antibodies were detected by horseradish peroxidase (HRP)-conjugated goat anti-human IgG Ab (Sigma-Aldrich) at a 405 nm absorbance using an ELISA plate reader (Tecan).

### Immunofluorescence assay

The immunofluorescence assay (IFA) was performed as described previously^23^. First, HeLa cells were grown on glass slides as monolayers. Next, the pCDNA 3.1 vector expressing HA from H7N9-AH/H7N9-SH1 was transfected into cells using Lipofectamine 2000 (Invitrogen). At 24 h post transfection, the slides were fixed with 4% paraformaldehyde and washed with PBS. The cells were then incubated with HNIgGA6 at 37 °C for 1 h. Bound antibodies were detected with a rhodamine-conjugated goat anti-human IgG antibody (Invitrogen) and were observed using a fluorescence microscope.

### Surface plasmon resonance binding

Surface plasmon resonance measurements were performed using a BIAcore 3000 instrument with CM5 chips (GE Healthcare) at room temperature (25 °C). All proteins were exchanged into PBST buffer (phosphate-buffered saline with 0.005% (v/v) Tween-20, pH 7.4) by gel filtration. The purified mAb was first immobilized on a CM5 chip. The HA1 proteins were serially diluted to between 0.078 and 2.5 nM (0.078, 0.156, 0.3125, 0.625, 1.25, and 2.5 nM) and were then flowed over the mAb. The binding kinetics were analyzed with BIAcore 3000 analysis software (BIAevaluation Version 4.1) using a 1:1 Langmuir binding model.

### H7N9 pseudovirus production and neutralization assay

To generate H7N9 pseudoviruses, viral HA- and NA-encoding sequences were cloned into the pcDNA 3.1 expression vector and were cotransfected with a pNL4–3-Luc-R-E- viral backbone plasmid into 293T cells using Lipofectamine 2000 (Invitrogen). Supernatants were collected 48 h after transfection. The H7N9 pseudovirus was purified by ultracentrifugation and dissolved in phosphate-buffered saline (PBS) before being stored at −80 °C in aliquots until their use in the neutralization assay. The 11 HAs were obtained by site-directed mutagenesis of the H7N9-AH HA-encoding gene according to the gene alignment results in Fig. 1b. The HA genes of three HPAI H7N9 variants, including A/Guangdong/17SF006/2017, A/Guangdong/Th005/2017, and A/Taiwan/1/2017, were custom synthesized and cloned into the pcDNA 3.1 vector. All the clones used were confirmed by sequencing. The viral titers were determined in MDCK cells by luciferase activity as the median tissue culture infective dose (TCID_50_). Neutralization assays were performed by incubating 100 TCID_50_ of pseudovirus with serially diluted antibodies at 37 °C for 1 h. The mixture was added to the cultured MDCK cells. Viral infection was quantified by the luciferase activity 48 h after infection. An irrelevant human IgG protein (anti-HIV, Immune Technology) was used as a negative control.
